# Involvement of the ACE2/Ang-(1–7)/MasR Axis in Pulmonary Fibrosis: Implications for COVID-19

**DOI:** 10.3390/ijms222312955

**Published:** 2021-11-30

**Authors:** Taylor Morganstein, Zahraa Haidar, Joshua Trivlidis, Ilan Azuelos, Megan Jiaxin Huang, David H. Eidelman, Carolyn J. Baglole

**Affiliations:** 1 Department of Pharmacology & Therapeutics, McGill University, Montreal, QC H3G 1Y6, Canada; taylor.morganstein@mail.mcgill.ca (T.M.); megan.huang@mail.mcgill.ca (M.J.H.); 2 Meakins-Christie Laboratories, Research Institute of the McGill University Health Centre (RI-MUHC), Montreal, QC H4A 3J1, Canada; zahraa.haidar@mail.mcgill.ca (Z.H.); joshua.trivlidis@mail.mcgill.ca (J.T.); ilan.azuelos@mcgill.ca (I.A.); david.h.eidelman@mcgill.ca (D.H.E.); 3 Translational Research in Respiratory Diseases Program at the RI-MUHC, Montreal, QC H4A 3J1, Canada; 4 Department of Medicine, McGill University, Montreal, QC H4A 3J1, Canada; 5 Department of Pathology, McGill University, Montreal, QC H3A 2B4, Canada

**Keywords:** pulmonary fibrosis, ACE2, COVID-19, SARS-CoV-2, cigarette smoke, cannabis

## Abstract

Pulmonary fibrosis is a chronic, fibrotic lung disease affecting 3 million people worldwide. The ACE2/Ang-(1–7)/MasR axis is of interest in pulmonary fibrosis due to evidence of its anti-fibrotic action. Current scientific evidence supports that inhibition of ACE2 causes enhanced fibrosis. ACE2 is also the primary receptor that facilitates the entry of SARS-CoV-2, the virus responsible for the current COVID-19 pandemic. COVID-19 is associated with a myriad of symptoms ranging from asymptomatic to severe pneumonia and acute respiratory distress syndrome (ARDS) leading to respiratory failure, mechanical ventilation, and often death. One of the potential complications in people who recover from COVID-19 is pulmonary fibrosis. Cigarette smoking is a risk factor for fibrotic lung diseases, including the idiopathic form of this disease (idiopathic pulmonary fibrosis), which has a prevalence of 41% to 83%. Cigarette smoke increases the expression of pulmonary ACE2 and is thought to alter susceptibility to COVID-19. Cannabis is another popular combustible product that shares some similarities with cigarette smoke, however, cannabis contains cannabinoids that may reduce inflammation and/or ACE2 levels. The role of cannabis smoke in the pathogenesis of pulmonary fibrosis remains unknown. This review aimed to characterize the ACE2-Ang-(1–7)-MasR Axis in the context of pulmonary fibrosis with an emphasis on risk factors, including the SARS-CoV-2 virus and exposure to environmental toxicants. In the context of the pandemic, there is a dire need for an understanding of pulmonary fibrotic events. More research is needed to understand the interplay between ACE2, pulmonary fibrosis, and susceptibility to coronavirus infection.

## 1. Introduction

Interstitial lung disease (ILD) refers to a heterogeneous collection of more than 200 lung disorders that are characterized by varying degrees of fibrosis and inflammation of the lung parenchyma or interstitium [1,2]. ILDs are divided into those with known causes, such as drugs and certain occupational/environmental exposures, and those with unknown causes such as idiopathic pulmonary fibrosis (IPF) [3]. IPF is a chronic, fibrotic lung disease affecting 3 million people worldwide, with a prognosis worse than many types of cancer [4,5]. Although the pathogenesis of IPF is not well understood, it is thought that the differentiation of fibroblasts to myofibroblasts followed by extracellular matrix (ECM) production is key to the disease, which scars the lungs. Cigarette smoking is the main risk factor for chronic obstructive pulmonary disease (COPD) and lung cancer but is also associated with the development of many types of ILDs, including IPF [6]. Other inhaled toxicants of concern include e-cigarettes and cannabis. Cannabis is the second most-used smoke product after tobacco [7]. Following the legalization of cannabis, approximately 75% of Canadians who use cannabis for recreational or medical purposes choose smoking as their preferred method of consumption. The pyrolysis of cannabis produces hundreds of chemicals. Similar to tobacco smoke, cannabis smoke contains carcinogens (e.g., polycyclic aromatic hydrocarbons {PAHs}) and other toxicants such as carbon monoxide [8]. Although the association of cigarette smoking on lung fibrosis development is described, there is minimal information on the relationship between cannabis smoke and fibrotic lung disease. Cannabis contains secondary metabolites called cannabinoids, such as Δ^9^-tetrahydrocannabinol (THC), which have the potential to reduce fibrosis through decreased TGF-β production [9]. Nevertheless, it is also plausible that cannabis smoke has similar effects to cigarette smoke in terms of IPF pathogenesis. 

There may also be interplay between inhalation exposures, pulmonary fibrosis, and the ACE2/Ang-(1–7)/MasR axis. This counter-regulatory axis, which is a part of the renin-angiotensin system (RAS), has anti-fibrotic and anti-proliferative abilities that may be relevant in pulmonary fibrosis [10]. However, this is further complicated by the fact that ACE2 is the cellular entry point for SARS-CoV-2 [11], the virus responsible for the current COVID-19 pandemic. Pulmonary ACE2 is found in type II alveolar cells and bronchial and tracheal epithelial cells. Changes in the expression of ACE2 may contribute to the susceptibility of SARS-CoV-2 infection and environmental factors such as smoking are now linked to an increase in ACE2 expression. Although controversial, high ACE2 expression may contribute to the risk of severe COVID-19 in certain populations, including those with underlying health conditions linked to smoking such as chronic obstructive pulmonary disease (COPD) [12,13]. Even among COVID-19 patients who recover, there is the potential for post-COVID-19 complications, including the development of pulmonary fibrosis [14,15]. In this review, we will investigate the relationship between the ACE2-Ang-(1–7)-MasR axis, pulmonary fibrosis, and the SARS-CoV-2 virus. Though each of these is well-characterized on its own, there lacks a global scientific understanding of how they impact one another in disease states, including coronavirus infection. 

## 2. Pulmonary Fibrosis

### 2.1. Overview

Upwards of 45% of deaths in industrialized countries are due to fibrotic diseases of which renal, hepatic, and pulmonary are the most common [16]. Pulmonary fibrosis is characterized by permanent destruction of the lung architecture due to scar formation. This scarring stiffens the lung and disrupts oxygen exchange, causing death from respiratory failure. Causes for fibrosis include a variety of environmental, occupational, and medication-related exposures or may result from systemic autoimmune or connective tissue diseases [17]. IPF is a form of fibrotic lung disease of unknown etiology and although the majority of IPF patients have a history of cigarette smoking, several other risk factors have been linked with the disease such as gastroesophageal reflux, chronic viral infections (Epstein Barr virus or hepatitis C), and familial history of ILD [18].

### 2.2. Risk Factors

Various environmental, microbial, and genetic factors have been proposed to play important roles in pulmonary fibrosis pathobiology [19]. Several risk factors are thought to contribute to the development of the fibrotic process by driving repeated micro-injury to the alveolar epithelium. Subsequently, lung cells develop aberrant behaviors in the repair process leading to the development and sustainment of the fibrotic process [20]. Cigarette smoke as well as other inhaled pollutants, such as metal and wood dust, have been associated with the risk of developing pulmonary fibrosis [19]. Viral, fungal, and bacterial microbial agents have been linked as potential risk factors as well [21]. Susceptibility appears to arise from several abnormal genetic features such as gene variants and transcriptional changes that may play a role in the loss of epithelial integrity of the lung [20]. Two large genome-wide association studies (GWAS) have reported common genetic variants that are important in telomere biology, host defense, and cellular barrier function. These genetic mutations play a crucial role in destabilizing the alveolar epithelium [22,23]. Evidence that other inhaled toxicants, including e-cigarette aerosols, alter the risk to the development of fibrotic lesions has been observed in some animal models as well [24].

### 2.3. Pathogenesis

Fibrosis likely evolves over many years prior to diagnosis where there is a modified lung structure, epithelial cell hyperplasia, dense fibrosis, and abnormal proliferation of mesenchymal cells [20]. Historically, pulmonary fibrosis was believed to result from chronic inflammation. The shift in paradigm that IPF, in particular, is not an inflammatory disease is largely based on (1) the presence of minimal signs of inflammation on lung biopsy and (2) the fact that immunosuppressive and corticosteroid therapies in treating IPF patients were harmful [25]. As such, the rejection of the “inflammation-driven fibrosis” hypothesis has led to the “fibrogenesis” theory [26]. It is now thought that lung fibrosis is an epithelium-driven disease due to recurrent micro-injuries that lead to impaired regeneration of epithelial tissue. Here, an unknown genetic mutation(s) predisposes an individual to develop lung fibrosis in addition to chronic epithelial cell turnover in response to injury, which is coupled with environmental exposure to risk factors such as cigarette smoke [1]. The mechanism by which cigarette smoke causes pulmonary fibrosis is not clear, but it may cause epithelial injury and activate pro-fibrotic cascades [27,28]. Other potential ways in which cigarette smoke may affect fibrotic disease development include the induction of endoplasmic reticulum (ER) stress [29], epigenetic changes such as DNA methylation, and changes in microRNA (miRNA) expression [30].

Although fibrotic lung disease involves many cell types and molecular processes, it is thought that disease development involves excessive, sequential injury and/or aberrant wound healing of the alveolar epithelium, although the distal airway epithelium may also be involved [31]. This aberrant wound healing response is typified by the accumulation of myofibroblasts [32]. Myofibroblasts are contractile cells that are highly resistant to apoptosis and produce copious amounts of ECM proteins such as collagens (COL) and fibronectin (FN) [33]. There are several possible sources of myofibroblasts, the predominant one being resident lung fibroblasts that differentiate into myofibroblasts. A second possible source occurs when epithelial cells differentiate to myofibroblasts by a process called epithelial-to-mesenchymal transition [34,35]. Myofibroblasts may also come from fibrocytes, a monocyte-derived circulating progenitor of fibroblasts [36,37]. Lastly, pericytes (microvascular mural cells) may adopt myofibroblast properties during the development of pulmonary fibrosis [38]. 

**Figure 1:**
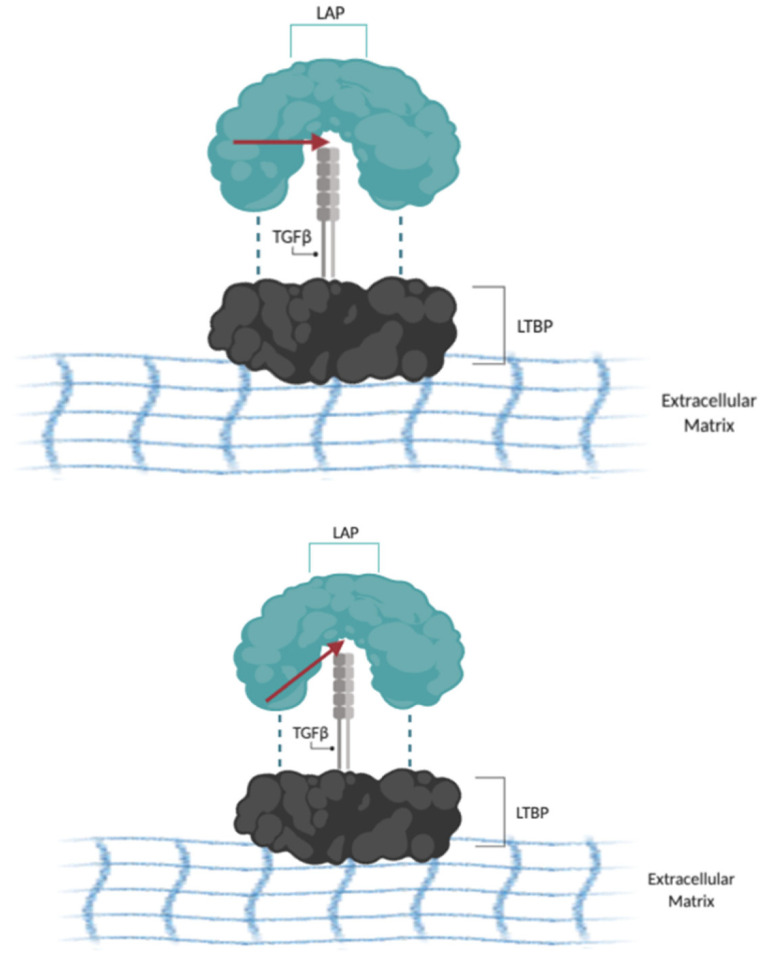
The TGFβ1 large latent complex (LLC): The LLC is made up of the LAP (blue), TGFβ1 (grey), and LTBP (black). The LAP is linked to the LTBP by disulfide linkages (dotted lines). As a result of contraction mediated by ανβ6 integrin, LAP and TGFβ1 can be proteolytically separated (red arrow). Dissociation of LAP from TGFβ1 allows for the endogenous release of TGFβ1. Information adapted from [48].

Regardless of their cellular source, myofibroblasts drive the fibrotic phenotype, as their excess matrix deposition may lead to pathologic lung fibrosis and remodeling [39]. This differentiation into myofibroblasts is directed by cytokines and growth factors, including platelet-derived growth factor (PDGF), vascular endothelial growth factor (VEGF), fibroblast growth factor (FGF), and transforming growth factor-β (TGF-β) [40]. One of the main sources of TGF-β in the lung is type II alveolar epithelial cells, which release TGF-β upon injury [41]. TGF-β then promotes the recruitment, differentiation, and survival of myofibroblasts [42,43,44]. Levels of TGF-β are significantly increased in the lungs of IPF patients [45] and experimentally, over-expression of active TGF-β in animal models induces lung fibrosis [46], supporting its role as a key driver of the fibrotic phenotype. TGF-β is secreted in its inactive form and can be activated by the αVβ6 integrin, leading to fibroblast-to-myofibroblast differentiation [47]. Under normal circumstances, inactive TGF-β is bound to a latency-associated peptide (LAP) which is crosslinked by disulfide bonds to the latent TGF-β binding protein (LTBP) and covalently bound to the ECM [48]. This is known as the TGF-β large latent complex (LLC) and acts to keep TGF-β in an inactive state (Figure 1). In lung fibrosis, type II AECs express increased levels of ανβ6 integrin, which can bind to LAP and release TGFβ1 [49]. Alongside mediators that induce contraction of epithelial cells such as thrombin, sphingosine 1-phosphate, and lysophosphatidic acid, type II AECs pull on the TGF-β that is bound to the ανβ6 integrin, thereby activating the chemokine. Another source of TGF-β is the unfolded protein response (UPR) that arises from the dysfunction of type II AECs [50].

There are three isoforms of TGF-β expressed in mammalian tissue: TGF-β1, TGF-β2, and TGF-β3. TGF-β1 is the predominant isoform expressed by most cells [50,51], but all isoforms exert their cellular effects by binding to the same high-affinity cell surface TGF-β type I and type II receptors [50,52]. Active TGF-β first binds to TβRII which recruits and phosphorylates TβRI. This induces a conformational change in TβRI, allowing it to then phosphorylate Smad proteins. Smads are latent cytoplasmic transcription factors and major TGF-β signal transducers [50,52,53]. There are nine Smad proteins in mammals that are characterized as receptor-regulated (R-Smads), common-partner co-Smad, and inhibitory Smads. Smad2/3 are the major R-Smads. After phosphorylation by TβRI, Smad2/3 dissociates from the receptor to form a heterotrimeric complex with the co-Smad Smad4. This complex (Smad2/3 and Smad4) then translocates to the nucleus to co-operate with other transcription factors and co-regulators to induce *ACTA2*, *COL1A1*, *COL3A1*, *FN*, etc., thereby increasing myofibroblast differentiation and ECM production. Non-canonical (i.e., non-SMAD) pathways include signaling through mitogen-activated protein kinases (MAPKs), Src kinases, and JAK2 (Janus kinase-2); these pathways can also be activated by risk factors for pulmonary fibrosis (e.g., cigarette smoke) [54] and converge on the signal transducer and activator of transcription 3 (STAT3), a transcription factor involved in the wound healing response [55]. Ultimately, TGF-β is involved in a plethora of profibrotic responses including epithelial cell apoptosis; epithelial-mesenchymal transition (EMT); epithelial cell migration; production of other profibrotic mediators; circulation of fibrocyte recruitment; and fibroblast activation, proliferation, and transformation into myofibroblasts [40]. However, the exact mechanism(s) leading to the development of pulmonary fibrosis is still not understood.

## 3. The ACE2/Ang-(1–7)/MasR Axis

The renin-angiotensin system (RAS) is a master regulator of many physiological processes including blood pressure and fluid balance. The human *ACE2* gene is located on chromosome Xp22 and contains 18 exons. Acting as a typical zinc metallopeptidase, the ACE2 protein is a type I integral membrane glycoprotein containing a single catalytic domain [56]. ACE2 has different physiological roles that focus on its trivalent functions as a negative regulator of the RAS, facilitator of amino acid transport, and receptor of the severe acute respiratory syndrome-coronavirus (SARS-CoV) and SARS-CoV-2. ACE2 is expressed in the cardiovascular system, kidneys, and testes, and is also broadly distributed in the liver, intestine, central nervous system, and lungs [57,58]. Pulmonary ACE2 expression is of great interest as the primary target organ of SARS-CoV-2 infection. Recent studies using single-cell RNA sequencing in humans revealed 0.64% of cells in lungs expressed ACE2 and 83% of ACE2 was enriched on type II AECs. Other types of lung cells, such as type I AECs, airway epithelial cells, endothelial cells, fibroblasts, and macrophages also express ACE2 [59,60].

ACE2 hydrolyzes AngII to Ang-(1–7) and thus is part of the ACE2/Ang-(1–7)/MasR axis [57]. Ang-(1–7) activates the Mas receptor (MasR); activation of MasR counteracts the effects of the binding of Ang II to Ang II receptor type 1 (AT1R), including vasoconstriction, enhanced inflammation, and thrombosis [61,62]. ACE2 can also convert Ang I to Ang-(1–9), a less-bioactive peptide [63]. The catalytic efficacy of ACE2 is 400-fold higher on Ang II than on Ang I [64]. Ang-(1–7)/MasR leads to the release of nitric oxide, prostaglandin E2, and bradykinin [65], resulting in vasodilation, natriuresis, and a decrease in oxidative stress and inflammation [66,67]. Thus, in the healthy lung, ACE2 expression serves various functions for cell types within the lungs. For example, ACE2 degradation of Ang II, which is proapoptotic for lung alveolar epithelial cells, may be acting as a survival factor [68]. Because Ang II increases collagen synthesis in lung fibroblasts, ACE2 can counter-regulate the wound healing response [69]. Finally, ACE2 is also cleaved by proteases such as the transmembrane serine protease 2 (TMPRSS2) and metalloproteinase domain-containing protein 17 (ADAM17) to yield a soluble form [70].

### Fibrosis and the ACE2/Ang-(1–7)/MasR Axis

Overall, the ACE2-Ang-(1–7)-MasR axis has anti-inflammatory and antifibrotic effects [14]. Studies have observed a decrease in pulmonary ACE2 during lung injury, but that a rescue from injury could be observed when ACE2 is overexpressed [57]. In models of lung inflammation, Ang-(1–7) decreases neutrophil and lymphocyte infiltrates, reduces perivascular and peri-bronchial inflammation, and prevents subsequent fibrosis [71,72]. Over-expression of ACE2 reduced bleomycin-induced fibrosis and hypertension [10]. Conversely, ACE2 is higher in IPF cells, particularly lung fibroblasts [60]. The fibrosis-reducing effect of ACE2 is thought to be due to its ability to degrade Ang II, which is profibrotic. Studies have shown that over-expression of Ang-(1–7) reduces TGB-β levels and the development of fibrosis [10,73]. Further, there is a decrease in α-SMA (a marker of fibroblast differentiation) upon treatment with Ang-(1–7) [74]. TGF-β also acts on the axis and has been shown to inhibit the expression of MasR, rendering Ang-(1–7) incapable of exerting its anti-fibrotic effects [75].

## 4. Coronavirus Disease-19 (COVID-19)

ACE2 is also the entry receptor for the novel β-coronavirus SARS-CoV-2, which is responsible for the ongoing COVID-19 pandemic. December 2019 marked the start of the COVID-19 pandemic, which originated in Wuhan, Hubei province, China [76]. At the time, 90% of cases were contained within Hubei province but quickly spread to various countries including Italy, the United States, Spain, India, and France [76,77]. To date, there are hundreds of millions of confirmed cases of COVID-19 across the globe, and millions of deaths (https://www.who.int/emergencies/diseases/novel-coronavirus-2019, accessed on 22 November 2021). The hallmark symptoms include fever, cough, and shortness of breath; however less common clinical presentations include dizziness, nausea, diarrhea, and loss of smell and taste [76,77,78]. Symptoms typically occur after an incubation period of 1–14 days, with a large majority of patients developing symptoms within 11.5 days (4,5). Long-term health effects include acute myocardial injury, chronic damage to the cardiovascular system, acute respiratory stress syndrome (ARDS), and shock [76]. 

COVID-19 is caused by the β-coronavirus belonging to the Coronaviridae family and is known as severe acute respiratory syndrome coronavirus-2 (SARS-CoV-2). Much of what is known about SARS-CoV-2 stems from the SARS-CoV outbreak of 2002, as the two viruses have significant structural homology and a 95–100% similarity between many of their proteins. SARS-CoV-2 is an enveloped coronavirus that consists of four main structural glycoproteins: spike (S), membrane (M), envelope (E), and nucleocapsid (N). The M, E, and N proteins are important for viral particle assembly and release, whereas the S protein is responsible for entry into host cells [79]. ACE2 has been unanimously accepted as the receptor for SARS-CoV-2, demonstrated by the lack of virus present in lung tissue upon ACE2 knockout. The binding affinity of SARS-CoV-2 is 10 to 20-fold stronger than for SARS-CoV, causing a graver disease progression. During infection, ACE2 is internalized, effectively reducing ACE2 levels at the cell surface [80]; infection can also cause the shedding of membrane-bound ACE2 into a soluble form whose function is still poorly understood [81,82]. The SARS-CoV-2 virus is highly infectious and has demonstrated a continued spread with a basic reproduction number (Ro) of 2.2–2.6 [76]. 

Several factors increase the risk of developing severe illness after infection with the SARS-CoV-2 virus, including age and underlying chronic medical conditions including cancer, kidney disease, COPD, cardiovascular disease, obesity, type 2 diabetes mellitus, and conditions causing an immunocompromised state [83]. The presence of underlying medical conditions increases the fatality rate from 0.9% to 10.5% [76]. There also exists a spectrum of disease severity that ranges from asymptomatic, mild, moderate, severe, and critical disease, with the majority of cases (81%) being mild to moderate. In both Canada and the U.S., between 16% and 19% of confirmed COVID-19 cases have been hospitalized, where 20% required Intensive Care Unit (ICU) intervention. While vaccines and treatments offer possible emergence from the COVID-19 pandemic, post-COVID-19 complications in individuals who have recovered from the disease have arisen. Among these, fibrosis has been noted as a major complication following infection [84] and is now recognized as a continuation of ARDS where there is a failure of re-epithelialization, fibroblast activation, and excessive ECM deposition [85]. 

### Pulmonary Fibrosis in COVID-19

To date, the long-term clinical consequences of SARS-CoV-2 remain unclear, although there are presentations in the literature outlining the development of fibrotic lung disease [86] where pulmonary fibrosis may manifest in severe or critical COVID-19 [87]. A meta-analysis found that 15% of COVID-19 patients developed ARDS, which is a risk factor for secondary pulmonary fibrosis [88]. Additional risk factors for pulmonary fibrosis following SARS-CoV-2 infection include illness severity, length of ICU stay, use of mechanical ventilation, smoking and chronic alcoholism, and other underlying diseases [89,90]. However, the most prominent factor is advanced age [89]. Data also suggests that COVID-19 severity is worsened by underlying IPF [87]. Since the risk factors for severe COVID-19 infection are shared with IPF, this group of patients could be at an increased risk of severe case presentations [87]. There is currently no biomarker to identify which patients will develop pulmonary fibrosis [91]. Several small-scale studies were conducted in which chest CT scans can observe fibrotic changes, including ground-glass opacities, linear opacities, interlobular septal thickening, reticulation, honeycombing, and bronchiectasis; in this study, 80% of patients had pulmonary fibrosis at discharge [92]. Notably, the incidence was low for patients with moderate COVID-19 but present in all patients with severe or critical cases. Currently, there are no treatments for COVID-19-induced pulmonary fibrosis [84]. 

**Figure 2:**
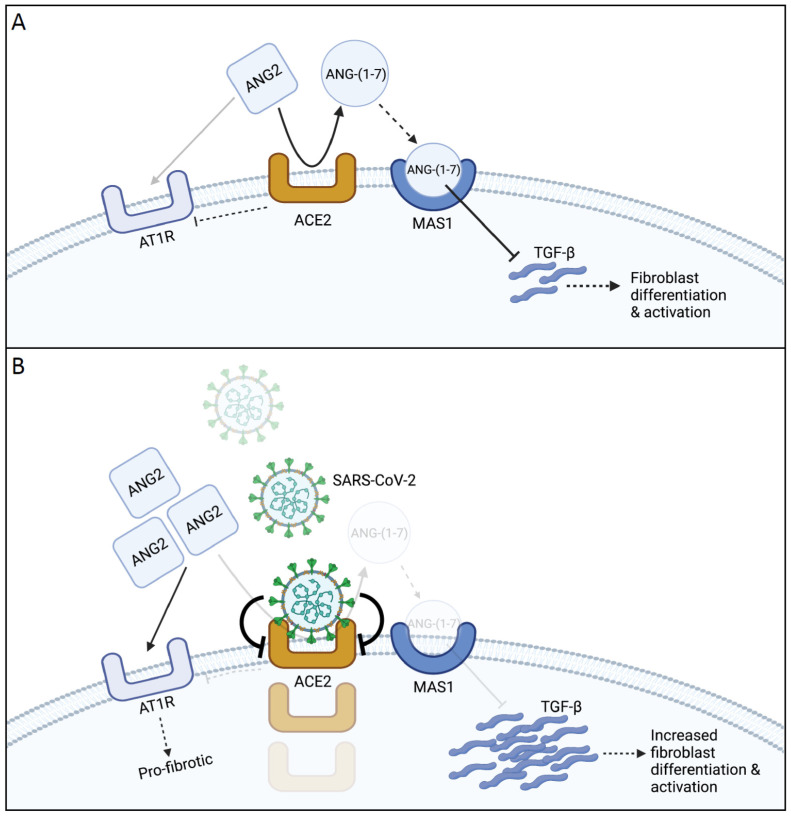
Overview of the ACE2/Ang-(1–7)/MasR Axis: (**A**) Healthy State (Anti-Inflammatory): In a healthy state, ACE II (ANG2) cleaves ANG II to ANG-(1-7) in a controlled balance against angiotensin receptor 1 (AT1R). TGF-β is responsible for fibroblast to myofibroblast differentiation and activation. ANG-(1-7) sequesters TGF-β, thus decreasing fibroblast differentiation. Increases in ANG-(1-7) maintain high levels of ACE2 and MasR (MAS1). (**B**) Disease State (Inflammatory): In the disease state, SARS-CoV2 binds ACE2, causing internalization and downregulation. Decreased levels of ACE II result in decreased ANG-(1-7), allowing TGF-B concentrations to rise. The increased TGF-beta, along with increased ANG II, causes increased fibrosis development.

Mechanistically, lung injury caused by SARS-CoV-2 is thought to lead to repair attempts by fibroproliferation and lung remodeling [89] (Figure 2). This may be mediated by an increase in TGF-β signaling, as in SARS-CoV-1 infection [93]. Injury during SARS-CoV-1 infection occurred in phases where the acute phase presented edema and alveolar shedding [75]. In the following phase, during weeks 2–5, there were signs of fibrosis and infiltration of inflammatory cells and fibroblasts in the alveolar spaces. During the final stages in weeks 6–8, fibrotic lung tissue with collagen deposits were observed [75]. In addition to TGF-β, other cytokines such as IL-6, CRP, and TNF-α are increased in COVID-19 patients [92,94]. Notable among the cytokines is interferon-γ (IFN-γ), where a recent study revealed that plasma IFN-γ levels could be indicative of pulmonary fibrosis development; decreased levels of IFN-γ corresponded with increased lung fibrosis at discharge [95]. There is conflicting information on the role of IFN-γ as it related to ACE2 expression, with studies showing both up and down regulation of ACE2 by IFNs [96,97]. While this suggests a possible link between cytokines, SARS-CoV-2, and fibrotic lung disease, the mechanistic underpinnings remain unclear.

## 5. Modulation of Pulmonary ACE2 Expression

Several observations may explain the increased incidence of pulmonary fibrosis in recovered COVID-19 patients. This includes the binding of SARS-CoV-2 to ACE2, which causes internalization and downregulating of its expression; this would cause an imbalance in the axis and an increasing concentration of the fibrosis-promoting Ang II. This downregulation of ACE2 may also partially explain the cytokine storm associated with COVID-19 by causing an imbalance in the Ang II/AT1R branch of the RAS [98]. Additionally, when examining the SARS-CoV virus, the infection caused expression of TGF-β in lung cells and enhanced signaling activity. Put together with data on SARS-CoV-2, it is plausible that the exacerbated fibrosis is due to increased TGF-β levels which are able to have a stronger action due to the lack of Ang-(1–7), as ACE2 is downregulated. Thus, perturbation of the RAS by infection and other underlying conditions/risk factors may underly susceptibility to post-COVID fibrosis.

### 5.1. Modulation of ACE2 by Cigarette Smoke

Cigarette smoke is a risk factor for pulmonary fibrosis, and emerging evidence supports that cigarette smoke also increases susceptibility to SARS-CoV-2 infection. In experimental studies using a preclinical cigarette smoke exposure model, investigators report that although there was no change in pulmonary ACE2 after an acute three-day cigarette smoke exposure, there was a significant increase after chronic exposure [60]. A separate study also observed a dose-dependent increase in ACE2 expression in murine lungs after chronic exposure [99]. The highest expression levels of ACE2 were observed from human patients who smoked, even when controlling for age, sex, race, and BMI [99]. Further, quitting smoking for 12 months resulted in a 40% decrease in ACE2 expression, suggesting that the induction of ACE2 by cigarette smoke may be reversible [99]. The induction of ACE2 expression occurred on the apical surface of bronchial epithelial cells which was reduced after long-term smoking cessation [100]. Several pathways are implicated in the regulation of ACE2 by smoke, including HIF-1α, an oxidative stress-induced transcription factor [100], and the α7-nicotinic acetylcholine receptor (nAChR) [101]. However, not all studies show increased ACE2 by smoke, as a recent study showed decreased ACE2 [102]. The implications for increased ACE2 in the context of lung diseases associated with smoking, such as COPD, are not clear but may be a compensatory mechanism to combat the heightened inflammatory response to smoke. Why cigarette smoke is also a risk factor for fibrosis despite upregulation of ACE2 is not clear, but could be due to the dysregulation of several cellular pathways by smoke in which RAS is unable to compensate. In the context of COVID-19 and the development of fibrotic lung disease, higher ACE2 levels induced by smoke may facilitate viral entry/infection, leading to ACE2 downregulation and an imbalance in RAS; pathologically, this is typified by inflammation and pneumonia followed by aberrant repair and extensive fibrosis. However, the mechanistic basis through which smoke regulates ACE2 expression is not established. 

### 5.2. Modulation of ACE2 by Other Inhalation Toxicants

Given the ability of cigarette smoke to modulate ACE2 levels, it is of interest to consider the potential impact of other inhaled toxicants on ACE2 levels. One such exposure is the inhalation of e-cigarette aerosols. E-cigarettes consist of a rechargeable battery, an atomizer (or heating element/coil), and a liquid that contains a solvent (usually propylene glycol {PG} and vegetable glycerin {VG}), nicotine, and various additives including flavors; e-cigarettes do not contain tobacco, but function to deliver nicotine to the brain. The use of e-cigarettes has become increasingly popular, especially among youth and young adults [103]. There is conflicting information on e-cigarette use and ACE2 expression. Analysis of a human dataset revealed that cigarette smoking increased ACE2 expression while the use of e-cigarettes did not [104]. Conversely in a murine model, there was an up-regulation in ACE2 after chronic exposure to nicotine-containing e-cigarettes [104]. Further studies are required to understand the impact of e-cigarette aerosol exposure on pulmonary ACE2 levels.

Another exposure with the potential to alter ACE2 expression is cannabis (also known as marijuana). Cannabis is the second most-used smoke product after tobacco [7]. According to the World Health Organization (WHO), around 150 million people (3% of the world population) consume cannabis each year, making this the most widely-used illicit drug in the world [105]. The most common way to consume cannabis is by inhalation of smoke from a joint or water pipe [106]. Similar to tobacco smoke, burning cannabis produces hundreds of chemicals, including carcinogens and other toxicants [8]. Both cannabis and cigarette smoke contain varying levels of hydrogen cyanide, aromatic amines, and polycyclic aromatic hydrocarbons (PAHs). Reports of lung damage, including fibrosis, were noted in cannabis smokers [107,108] and have been reported in experimental studies performed on primates [109]. Yet the contribution of cannabis smoke in the pathogenesis of pulmonary fibrosis remains largely unknown. 

However, cigarette and cannabis smoke also differ because of their unique secondary metabolites, with tobacco containing nicotine and cannabis containing cannabinoids such as Δ^9^-tetrahydrocannabinol (THC) and cannabidiol (CBD). Cannabinoids themselves may have anti-fibrotic effects. For example, CBD is a ligand for the peroxisome proliferator-activated receptor (PPAR)-γ, a receptor that can regulate myofibroblast differentiation in various organs [110,111]. Moreover, Δ^9^-tetrahydrocannabinolic acid (Δ^9^-THCA), the non-psychotropic precursor of Δ^9^-THC can also prevent TGF-β-induced liver fibrosis [112]. Although the effects of cannabinoids on lung fibrosis are not clear, a recent study showed that activation of cannabinoid receptor 2 (CB2) protects against bleomycin-induced fibrosis [113].

To date, there is also very little research on the interaction of ACE2, cannabinoids, and cannabis smoke, although there is evidence that CBD decreased ACE2 expression [114], may inhibit SARS-CoV-2 replication [115] and reduced COVID-19 related inflammation [116]. Studies utilizing over 800 *C. sativa* strains in 3D human models of COVID-19 target tissues (oral, airway, and intestinal) noted that high CBD/low THC extracts downregulate ACE2 gene and ACE2 protein levels [114,117]. Further, these extracts downregulated TMPRSS2, a protein implicated in the SARS-CoV2 entry into cells [114]. CBD is of interest for lung fibrosis in COVID-19 for its ability to modulate myofibroblast differentiation as a peroxisome proliferator-activated receptor (PPAR)-γ ligand [118].

## 6. Conclusions

Our review examined the relationship between the ACE2/Ang-(1–7)/MasR axis, pulmonary fibrosis in the context of the COVID-19 pandemic. Although it has been widely accepted that the axis exerts an anti-fibrotic effect, this interaction with TGF-β suggests a mechanistic link. Since Ang-(1–7) levels are dependent upon cleavage of Ang II by ACE2, it becomes important to explore expression in relation to known risk factors (e.g., cigarette smoke) and other putative modulators (e.g., cannabis smoke and e-cigarette aerosols). Data in this field remains limited, and further studies are needed to conclude the effects of other toxicants on fibrosis development. It is thus necessary for additional research on other factors such as CBD to alter ACE2 expression and counter the deleterious effect of SARS-CoV-2 infection. Our review emphasizes the necessity for further research on each factor and its interplay.
